# Exploring Canadian perceptions and experiences of stigma during the COVID-19 pandemic

**DOI:** 10.3389/fpubh.2023.1068268

**Published:** 2023-03-07

**Authors:** Christine Fahim, Jeanette Cooper, Suvabna Theivendrampillai, Ba' Pham, Sharon E. Straus

**Affiliations:** Knowledge Translation Program, Li Ka Shing Knowledge Institute, Unity Health Toronto, Toronto, ON, Canada

**Keywords:** COVID-19, stigma, misinformation, health behaviors, Canada, discrimination

## Abstract

**Background:**

The COVID-19 pandemic has led to stigmatization of individuals based on race/ethnicity, age, gender, and occupation, among other factors. We canvassed Canadian residents to explore perceptions of and experiences with stigma during the COVID-19 pandemic.

**Methods:**

We conducted an online survey between June 10 and December 31 2020. The survey was rooted in the Health Stigma and Discrimination Framework and included multiple choice, Likert and open-ended questions related to *perceived* and *experienced* stigma. Residents of Ontario, Canada were eligible to participate and we aimed to recruit a sample that was diverse by race/ethnicity and age.

**Results:**

A total of 1,823 individuals participated in the survey (54% women, 39% men; 54% 18–40 years old, 28% 41–60 years old, 12% 61+ years old; 33% White, 26% East/SouthEast Asian, 14% Black, 12% South Asian). Fifty-one percent of participants agreed/strongly agreed that racist views had increased toward certain racial/ethnic groups in Canada during the pandemic. Participants perceived that people in Canada were stigmatized during the pandemic because of race/ethnicity (37%), political beliefs (26%), older age (24%), being a healthcare worker (23%), younger age (22%), being an essential worker (21%), and gender (11%). Thirty-nine percent of respondents feared experiencing and 37% experienced stigmatization during the pandemic. Men, individuals aged 18–40, and racialized participants were more likely to fear or experience stigma. With respect to health behaviors, 74, 68, and 59% of respondents were comfortable masking in public, seeking medical care if they became ill, and getting tested for COVID-19, respectively. Men were less likely to indicate comfort with mask wearing or seeking medical care. Participants aged 18–40 and Black participants were less likely to indicate comfort with all three behaviors compared to those over age 41 and White participants, respectively. South Asian participants were less likely to be comfortable seeking medical care compared to White Participants.

**Discussion:**

Participants feared or experienced stigmatization towards various demographic characteristics during the COVID-19 pandemic. It is critical that the factors driving stigma during health emergencies in Canada be better understood in order to develop effective public health messaging and interventions.

## Introduction

The COVID-19 pandemic led to rises in hate speech, racism, and stigmatization of certain populations (1–3). At the onset of the pandemic, this stigma was particularly directed toward people of Chinese (4–6) or East-Asian decent (4–6). However, as the pandemic evolved, other groups in Canada were stigmatized including those who had COVID-19, healthcare workers, individuals from communities perceived to have experienced outbreaks (particularly racialized communities), and individuals who did or did not participate in public health recommendations such as masking (7–9). Intersecting factors and ongoing structural inequities further resulted in the disproportionate impact of the pandemic on women, racialized persons, and frontline and healthcare workers (7).

Stigma can be driven by fears and a lack of knowledge (10). As the evidence on COVID-19 evolved, so did public health policies aimed at curbing its spread (11). These changing policies coupled with a perceived lack of transparency and limited public health information left individuals feeling a sense of distrust and confusion surrounding the pandemic (12). This created a climate for an “infodemic”, where public fear and desire for knowledge coupled with widespread misinformation facilitated the spread of stigmatization and discrimination (13–17).

Stigmatization can create a barrier to accessing health services and participating in public health behaviors (e.g., masking). Stangl et al., developers of the Health Stigma and Discrimination Framework (HSDF) outline that stigma “influences public health outcomes by worsening, undermining, or impeding a number of processes including social relationships, resource availability, stress, and psychological and behavioral responses, [thereby] exacerbating poor health” (10). The framework developers emphasize the importance of moving away from framing stigma as experiences between the individual “stigmatizer” vs. the “stigmatized” and rather, giving attention to narratives that drive stigma within broader organizational, community and historical systems that shape public opinion.

The purpose of this study was to use the HSDF to explore perceptions and experiences of stigma during the COVID-19 pandemic in Canada. Specifically, we aimed to explore perceptions and experiences of stigma among Ontario residents by gender, age, and race/ethnicity.

## Materials and methods

The study was approved by the Toronto Academic Health Science Network REB (REB#20-092).

### Study design

#### Survey study

The conduct and reporting of our survey study adhered to the Checklist for Reporting Results of Internet E-Surveys (CHERRIES) (18, 19). We aimed to recruit a convenience sample of participants who were residents of Ontario, Canada and reflected diversity with respect to age, gender and race/ethnicity.

#### Survey development

The survey was rooted in the HSDF which describes health stigmatization across a socio-ecological spectrum and considers how intersecting characteristics (such as race, sex, gender, age) interact within organizations, communities and public policy (18). The HSDF distinguishes between *stigmatized experiences* that impact outcomes (e.g., emotional health, social exclusion, access to treatment) and *stigma practices* (e.g., fear/misinformation that perpetuate stereotypes and discrimination), thus categorizing stigma as *perceived* and *experienced*.

The survey included 11 demographic questions and 36 questions on stigma. Survey questions included multiple choice, 5 point Likert scale, and open-ended responses (see Appendix A for survey). The survey was developed by the research team; team members piloted the survey for clarity prior to dissemination.

### Recruitment

We aimed to generate a sample that was evenly distributed by male and female gender (although we were inclusive of all gender categories) and evenly distributed by age ranges (>18 years). With respect to race/ethnicity, we aimed to generate a sample that included a minimum of 25% of respondents who self-identified as East Asian or Black, respectively. We also aimed to include representation by other races/ethnicities.

We conducted an open survey with multiple recruitment channels. Survey links were distributed *via* our community partners including the Chinese Canadian National Council for Social Justice and the Chinese Canadian National Council Toronto Chapter, which are advocacy organizations for social justice. Study recruitment ads were also posted to our websites (Knowledge Translation Program, SPOR Evidence Alliance, KT Canada, Unity Health Toronto) and to Twitter, LinkedIn, Kijiji, and Reddit. Additionally, we sent study invitations *via* email to community and student organizations representing Black, Indigenous, and racialized (BIPOC) populations. We also enlisted a market organization, Canadian Viewpoint (20), to disseminate our survey and support recruitment of a diverse Ontario population. Canadian Viewpoint distributed the survey using an email invitation to their email listservs, which were composed of individuals who previously agreed to receiving email communications from Canadian Viewpoint. Survey participants had the option of entering into a draw for the chance to win a Visa gift card.

### Informed consent

Participants who clicked on the survey link were directed to the survey landing page, which provided an overview of the study purpose, a description of how to complete the survey, and consent information. We provided a statement that consent would be implied by accessing the survey. All participation was voluntary; participants who did not wish to participate were directed to close the webpage. Responses were anonymous and were not mandatory.

### Data collection

Surveys were posted using Qualtrics on our program website and distributed by Canadian Viewpoint. Data were collected between June 10th 2020–December 31st 2020.

### Data analysis

Questionnaires with <5% data completion, completed by non-Ontario residents and duplicates were excluded from analysis. Quantitative data were summarized using descriptive statistics; textual responses were analyzed using content analysis by a team member experienced in qualitative analysis. Responses to the stigma questions were analyzed using ordinal regression to explore the association between stigma perceptions and experiences and demographic characteristics. We grouped responses to the 5-point Likert scale questions into three categories (Strongly disagree and Disagree; Neither Agree/Disagree; and Strongly Agree and Agree). We also combined groups by age and race/ethnicity to facilitate regression analyses. We excluded gender-diverse persons from the regression analysis by gender given insufficient sample size (<1% of the overall sample).

We used ordinal regression analysis conducted using the R statistical software (21), the R packages “polyr”, “foreign”, “ggplot2”, “MASS”, “Hmisc” and “reshape2” and RStudio (22). Statistical analysis was led by a statistician (BP).

## Results

### Participant demographics

A total of 1,823 individuals participated in the survey (Table 1). A total of 54.31% of participants identified their gender as female, 38.56% as male, 0.49% identified as genderfluid, transgender, gender variant, trans male, or non-binary and 6.64% did not respond. 53.87% of participants were between 18 and 40 years old, 28.41% were 41–60 years old and 11.79% were 61 years or older. With respect to race/ethnicity, 32.69% identified as White, 26% as East Asian or Southeast Asian, 14.21% as Black, and 11.68% as South Asian. 9.05% identified as LatinX, Middle Eastern, South American, Multiple Ethnicities, or Other and 5.48% of the sample identified as Indigenous. Approximately fifty percent of the sample were college/university educated, 13.55% had some college/university, and 18.43% had a post-graduate degree. Over half (52.06%) of participants worked full-time. The majority of the participants (82.99%) were Canadian citizens. Participants most commonly resided in Central Ontario (35.38%) and in the Toronto region (33.85%) (Table 1).

**Table 1:** Participant demographics.

**Demographic Characteristic**	***N* (1,823)**	**%**
**Gender**		
Female	990	54.31
Male	703	38.56
Prefer to Self-Describe: *Genderfluid, transgender, lesbian, gender variant, trans male, non-binary*	9	0.49
Prefer not to say	14	0.77
No Response	107	5.87
**Age (years)**		
18–40	982	53.87
41–60	518	28.41
61+	215	11.79
No response	108	5.92
**Race/Ethnicity** ^*^		
White	596	32.69
East or Southeast Asian	474	26.00
Black	259	14.21
South Asian	213	11.68
Others: *LatinX, Middle Eastern, South American, Multiple Ethnicities, Other*	165	9.05
No Response	116	6.36
**Indigenous identity**		
First nations	38	2.08
Inuit	3	0.16
Metis	30	1.65
Other	29	1.59
Does not Identify Indigenous	1,614	88.54
No response	109	5.98
**Education level**		
Up to 12th grade	38	2.08
High School/Equivalent	169	9.27
Some College/University	259	14.21
College/University degree	907	49.75
Post-Graduate degree	336	18.43
No response	114	6.25
**Employment status** ^*^		
Full-time	949	52.06
Part-time	247	13.55
Caregiver	33	1.81
Student	144	7.90
Seeking work	116	6.36
Retired	174	9.54
Other	143	7.84
No response	116	6.36
**Immigration status**		
Canadian citizen (born in Canada)	921	50.52
Canadian citizen (foreign born)	592	32.47
Permanent resident	142	7.79
Temporary resident/student visa	47	2.58
Other	6	0.33
No response	115	6.31
**Place of residence**		
Toronto region	617	33.85
Northern Ontario	53	2.91
Central Ontario	645	35.38
Eastern Ontario	172	9.43
Western Ontario	139	7.62
No response	197	10.81

^*^Participants could select more than one response.

### Perceptions of blame

In response to the question “Do you think there is someone to blame for COVID-19”, 54.91% of participants answered not at all/a little, 22.60% answered somewhat, and 18.98% answered a lot/quite a lot. In the open-ended responses, participants (*n* = 1,155) assigned blame to the following entities: China/Chinese government/health officials (36.36%), governments (general) and public health officials (16.62%), people who do not follow public health advice (e.g., anti-masking, not following social distancing; 9.52%), Donald Trump/the US government (4.16%), wet markets (4.16%), and people who consume animals (3.98). Other responses (<4% each) included: the World Health Organization, laboratories in which the virus may have originated, media, travelers, and scientists.

### Perceptions of stigma and racist views in Canada

51.45% of participants agreed/strongly agreed that racist views had increased toward certain racial/ethnic groups in Canada during the pandemic; 41.19% believed Canadians were speaking out against discrimination and stigmatization (Table 2). Men had slightly lower odds of agreeing with this statement compared to women participants (Table 3). East/South Asian and other non-White, non-Black, and non-East/South East Asian participants (grouped as “other”) were more likely to agree compared to White participants (OR 1.71, 1.85, respectively, Table 3).

**Table 2:** Proportion of participants that agreed/strongly agreed with statements.

**Statement**	**% of Participants who agreed/strongly agreed**
**Experiences/perceptions of racism during the pandemic**	
People have said things that were untrue about my race/ethnic group related to COVID-19	21.67%
I believe racist views have increased toward certain racial/ethnic groups in Canada during COVID-19	51.45%
I believe Canadians are speaking out against discrimination and stigmatization	41.20%
**Social interactions during the pandemic**	
I have experienced a slur/joke about my identity during COVID-19	16.78%
I have experienced rejection because of my beliefs about masking or social distancing during COVID-19	18.65%
People have acted as if they were afraid of me during COVID-19	22.98%
People have acted as if I am not smart during COVID-19	16.07%
People have threatened or harassed me during COVID-19	14.21%
People have treated me poorly at restaurants/stores during COVID-19	13.88%
**Fears experiencing stigmatization based on [demographic factor] during the pandemic**	
Sex	8.67%
Gender	8.56%
Race/Ethnicity	21.17%
Age	16.57%
Immigration status	11.46%
Level of ability	10.59%
Sexual orientation	8.17%
Level of education	9.65%
Religion	9.93%
Political view	13.88%
Place of work	13.93%
Don't fear stigmatization	32.25%
**Has experienced stigmatization based on [demographic factor] during the pandemic**	
Sex	7.18%
Gender	6.69%
Race/Ethnicity	18.16%
Age	17.06%
Immigration status	10.15%
Level of ability	10.59%
Sexual orientation	7.73%
Level of education	9.10%
Religion	9.10%
Political view	12.62%
Place of work	13.22%
**Comfort with public health behaviors during the pandemic**	
I have felt/would feel comfortable wearing a mask in public	73.67%
I have felt/would feel comfortable getting tested for COVID-19	59.19%
I have felt/would feel comfortable seeking medical care if I got sick during the pandemic	67.80%

**Table 3:** Associations between experiences with social interactions during the COVID-19 pandemic and demographic characteristics^1^.

**Statement**	**Gender**			**Age**					**Race**								
	**Female**	**Male**		**18–40**	**41–60**		**61**+		**White**	**Black**		**East** + **Southeast Asian**		**South Asian**		**Other**	
		**OR**	**CI**		**OR**	**CI**	**OR**	**CI**		**OR**	**CI**	**OR**	**CI**	**OR**	**CI**	**OR**	**CI**
**Experiences/perceptions of racism during the pandemic**																	
People have said things that were untrue about my race/ethnic group related to COVID-19	Reference	1.58	1.28–1.95^*^	Reference	0.55	0.43–0.69^*^	0.36	0.24–0.53^*^	Reference	4.10	2.94–5.73^*^	11.67	8.75–15.70^*^	3.92	2.74–5.62^*^	4.12	2.79–6.08^*^
I believe racist views have increased toward certain racial/ethnic groups in Canada during COVID−19		0.74	0.61–0.89^*^		1.01	0.82–1.25	0.82	0.62–1.11		1.26	0.95–1.68	1.71	1.35–2.19^*^	1.01	0.75–1.38	1.85	1.30–2.65^*^
**Social interactions during the pandemic**																	
I have experienced a slur/joke about my identity during COVID-19	Reference	1.46	1.18–1.81^*^	Reference	0.56	0.44–0.71^*^	0.32	0.21–0.48^*^	Reference	2.60	1.86–3.62^*^	4.95	3.74–6.58^*^	2.81	1.97–4.00^*^	2.34	1.57–3.47^*^
I have experienced rejection because of my beliefs about masking or social distancing during COVID-19		1.26	1.04–1.54^*^		0.64	0.51–0.80^*^	0.40	0.28–0.57^*^		1.55	1.14–2.10^*^	1.65	1.28–2.14^*^	1.58	1.13–2.20^*^	1.74	1.21–2.50^*^
People have acted as if they were afraid of me during COVID-19		1.37	1.13–1.65^*^		0.57	0.46–0.71^*^	0.39	0.28–0.54^*^		1.56	1.17–2.09^*^	2.30	1.81–2.93^*^	1.45	1.06–1.98^*^	1.36	0.95–1.93
People have acted as if I am not smart during COVID-19		1.64	1.34–2.01^*^		0.61	0.49–0.77	0.32	0.22–0.47^*^		1.81	1.32–2.47^*^	2.16	1.66–2.81^*^	1.96	1.40–2.72^*^	1.49	1.01–2.17^*^
People have threatened or harassed me during COVID-19		1.76	1.42–2.18^*^		0.58	0.45–0.73^*^	0.25	0.15–0.38^*^		1.95	1.39–2.74^*^	2.83	2.14–3.77^*^	2.31	1.57–3.40^*^	2.33	1.63–3.32^*^
People have treated me poorly at restaurants/stores during COVID-19		1.72	1.39–2.12^*^		0.54	0.42–0.68^*^	0.31	0.20–0.46^*^		2.25	1.63–3.10^*^	2.23	1.69–2.94^*^	2.47	1.74–−3.49^*^	1.85	1.25–2.72^*^

^1^Odds-ratio estimates of the response of agree/strongly agree with statements in the left column for different levels of the demographic variables.

^*^Denotes significant finding at α = 0.05.

Participants believed individuals in Canada had been stigmatized “a lot/quite a lot” during the pandemic due to: race/ethnicity (37.46%), political beliefs (25.78%), older age (23.53%), being a healthcare worker (22.65%), younger age (21.94%), being an essential worker (21.39%), and gender (10.75%).

### Fear of stigma

A total of 25.34% of participants said they were very afraid/concerned of experiencing stigma or racism, 32.86% were a little afraid/concerned and 28.96% were not afraid/concerned (4.06% were unsure, 5.16% selected N/A, and 3.62% did not respond). 21.67% of participants agreed that people had said untrue things about their race/ethnic group related to COVID-19; men were more likely to agree with this statement compared to women and racialized participants [particularly East or Southeast Asian participants (OR 11.67, Table 3)] were significantly more likely to agree compared to White participants.

40.10% percent of participants feared being stigmatized during the pandemic; 32.25% did not fear stigmatization (Table 2). Participants reported fearing stigmatization during the pandemic based on: race/ethnicity (21.17%), age (16.57%), political view (13.88%), place of work (13.93%), immigration status (11.46%), religion (9.93%), level of education (9.65%), level of ability (10.58%), sex (8.67%), gender (8.56%), and sexual orientation (8.17%).

Men were more likely to fear being stigmatized due to sex, gender, immigration status, level of ability, sexual orientation, level of education, religion, political view compared to Women (Table 4). Participants aged 18–40 were more likely to fear stigmatization on almost all assessed demographic factors compared to those over the age of 40 (Table 4). Racialized participants were more likely to report fear of stigmatization during the pandemic on all assessed demographic factors compared to White participants. Notably, East and Southeast Asian, Black, South Asian, and Other participants had an OR of 18.73, OR 6.28, OR 5.29, and OR 4.51 of fearing being stigmatized by race/ethnicity compared to White participants (see Table 4).

**Table 4:** Association between fear of being stigmatized based on demographic factors and demographic characteristics^1^.

**I fear being stigmatized during COVId-19 because of my [demographic factor]**	**Gender**			**Age**					**Race**								
	**Female**	**Male**		**18-40**	**41-60**		**61**+		**White**	**Black**		**East** + **Southeast Asian**		**South Asian**		**Other**	
		**OR**	**CI**		**OR**	**CI**	**OR**	**CI**		**OR**	**CI**	**OR**	**CI**	**OR**	**CI**	**OR**	**CI**
Sex	Reference	1.48	1.18–1.87^*^	Reference	0.69	0.53–0.90^*^	0.44	0.28–0.68^*^	Reference	3.04	2.12–4.38^*^	2.76	2.02–3.81^*^	3.39	2.32–4.96^*^	2.48	1.62–3.79^*^
Gender		1.45	1.15–1.83^*^		0.62	0.48–0.80^*^	0.42	0.27–0.65^*^		3.38	2.35–4.88^*^	3.05	2.22–4.22^*^	3.56	2.43–5.23^*^	2.89	1.88–4.41^*^
Race		1.00	0.81–1.24		0.59	0.46–0.75^*^	0.60	0.41–0.87^*^		6.28	4.45–8.93^*^	18.73	13.76–25.82^*^	5.29	3.66–7.68^*^	4.51	2.99–6.79^*^
Age		1.17	0.96–1.43		0.42	0.33–0.54^*^	0.95	0.69–1.30		2.21	1.63–3.00^*^	1.86	1.43–2.42^*^	1.86	1.33–2.60^*^	2.29	1.60–3.28^*^
Immigration status		1.46	1.17–1.82^*^		0.54	0.42–0.69^*^	0.42	0.28–0.64^*^		3.88	2.72–5.56^*^	5.24	3.85–7.20^*^	4.25	2.92–6.22^*^	3.16	2.07–4.81^*^
Level of ability		1.43	1.14–1.78^*^		0.68	0.53–0.87^*^	0.68	0.46–0.98^*^		2.55	1.82–3.58^*^	2.36	1.76–3.18^*^	2.59	1.80–3.70^*^	2.30	1.52–3.43^*^
Sexual orientation		1.72	1.36–2.17^*^		0.58	0.44–0.75^*^	0.34	0.21–0.54^*^		3.89	2.67–5.68^*^	3.65	2.63–5.13^*^	4.25	2.88–6.32^*^	3.00	1.92–4.67^*^
Level of education		1.40	1.12–1.75^*^		0.60	0.46–0.77^*^	0.38	0.24–0.57^*^		2.51	1.77–3.56^*^	2.42	1.79–3.28^*^	2.72	1.88–3.93^*^	2.01	1.31–3.05^*^
Religion		1.58	1.26–1.97^*^		0.52	0.40–0.67^*^	0.43	0.28–0.63^*^		3.57	2.53–5.07^*^	3.26	2.40–4.45^*^	3.85	2.67–5.58^*^	3.19	2.11–4.81^*^
Political view		1.46	1.18–1.80^*^		0.49	0.38–0.62^*^	0.31	0.20–0.45^*^		1.94	1.41–2.67^*^	2.12	1.62–2.79^*^	2.07	1.47–2.92^*^	2.02	1.38–2.96^*^
Place of work		1.13	0.92–1.39		0.54	0.43–0.69^*^	0.36	0.24–0.53^*^		2.00	1.45–2.75^*^	2.03	1.55–2.67^*^	1.92	1.36–2.71^*^	1.78	1.22–2.58^*^
I Don't fear stigmatization		1.24	1.03–1.49^*^		0.82	0.67–1.00	1.03	0.77–1.39		0.81	0.61–1.07	0.59	0.46–0.74^*^	0.85	0.63–1.15	1.08	0.78–1.50

^1^Odds-ratio estimates of the response of agree/strongly agree with statements in the left column for different levels of the demographic variables.

^*^Denotes significant finding at α = 0.05.

### Experiences of stigma

Table 2 highlights participants' experiences during the pandemic. Men and racialized participants were more likely to report experiencing negative social interactions during the pandemic compared to Women and White participants, respectively. Participants aged 18–40 were more likely to report negative social interactions compared to those over age 40, with one exception—Participants aged 40+ were more likely to report that people have acted afraid of them during the pandemic, compared to those 18–40 (Table 3).

Men were more likely to fear being stigmatized due to sex, gender, immigration status, level of ability, sexual orientation, level of education, religion, political view, and place of work compared to Women (Table 5). Participants aged 18–40 were more likely to fear stigmatization on all assessed demographic factors compared to those over the age of 40, with the exception of experiences of stigma based on age compared to those 61+ (OR 1.01, CI 0.74–1.38, Table 5). Similar to fear of stigma, racialized participants were more likely to report stigma experiences based on all assessed demographic factors compared to White participants.

**Table 5:** Association between experiences of being stigmatized based on demographic factors and demographic characteristics^1^.

**I have been stigmatized during COVID-19 because of my [demographic factor]**	**Gender**			**Age**					**Race**								
	**Female**	**Male**		**18–40**	**41–60**		**61**+		**White**	**Black**		**East** + **Southeast Asian**		**South Asian**		**Other**	
		**OR**	**CI**		**OR**	**CI**	**OR**	**CI**		**OR**	**CI**	**OR**	**CI**	**OR**	**CI**	**OR**	**CI**
Sex	Reference	1.73	1.36–2.19^*^	Reference	0.69	0.52–0.89^*^	0.33	0.19–0.54^*^	Reference	3.62	2.47–5.33^*^	3.61	2.54–5.10^*^	4.18	2.80–6.26^*^	2.00	1.22–3.22^*^
Gender		1.72	1.35–2.18^*^		0.68	0.52–0.89^*^	0.33	0.20–0.53^*^		3.29	2.26–4.81^*^	2.95	2.12–4.13^*^	3.91	2.64–5.80^*^	2.08	1.30–3.28^*^
Race		1.19	0.96–1.47		0.62	0.48–0.78^*^	0.41	0.27–0.62^*^		5.34	3.74–7.68^*^	14.9	10.88–20.63^*^	4.72	3.23–6.94^*^	3.97	2.60–6.06^*^
Age		1.11	0.91–1.36		0.45	0.35–0.56^*^	1.01	0.74–1.38		1.68	1.24–2.27^*^	1.47	1.13–1.90^*^	1.59	1.14–2.20^*^	1.70	1.18–2.42^*^
Immigration status		1.60	1.27–2.01^*^		0.64	0.49–0.82^*^	0.43	0.27–0.67^*^		3.62	2.50–5.27^*^	5.23	3.80–7.27^*^	4.17	2.82–6.17^*^	2.12	1.33–3.35^*^
Level of ability		1.64	1.31–2.06^*^		0.72	0.56–0.92^*^	0.54	0.36–0.81^*^		2.62	1.85–3.72^*^	2.38	1.76–3.24^*^	3.08	2.14–4.44^*^	2.07	1.35–3.14^*^
Sexual orientation		2.13	1.67–2.71^*^		0.67	0.50–0.87^*^	0.29	0.16–0.48^*^		3.66	2.48–5.45^*^	3.26	2.31–4.66^*^	5.19	3.47–7.80^*^	2.60	1.61–4.15^*^
Level of education		1.86	1.47–2.34^*^		0.62	0.48–0.81^*^	0.28	0.16–0.45^*^		2.94	2.05–4.21^*^	2.39	1.75–3.30^*^	2.78	1.89–4.08^*^	1.90	1.21–2.94^*^
Religion		1.81	1.43–2.29^*^		0.58	0.44–0.76^*^	0.35	0.21–0.55^*^		3.20	2.21–4.64^*^	2.93	2.12–4.08^*^	3.37	2.28–4.97^*^	2.46	1.57–3.82^*^
Political view		1.66	1.34–2.07^*^		0.51	0.40–0.65^*^	0.22	0.14–0.34^*^		1.49	1.06–2.07^*^	1.66	1.25–2.20^*^	1.76	1.24–2.50^*^	1.65	1.12–2.43^*^
Place of work		1.43	1.16–1.76^*^		0.54	0.42–0.68^*^	0.28	0.18–0.42^*^		1.82	1.32–2.50^*^	1.65	1.26–2.18^*^	1.81	1.28–2.56^*^	1.57	1.06–2.30^*^

^1^Odds–ratio estimates of the response of agree/strongly agree with statements in the left column for different levels of the demographic variables.

^*^Denotes significant finding at α = 0.05.

### Health behaviors

The majority of participants reported being comfortable with three common health behaviors of the COVID-19 pandemic; 73.67% felt comfortable wearing a mask in public, 67.80% were comfortable seeking medical care if they became ill during the pandemic, and 59.19% were comfortable getting tested for COVID-19 (Table 2). Men were less likely to indicate comfort with mask wearing or seeking medical care. Participants aged 18–40 and Black participants were less likely to indicate comfort with all three behaviors compared to those over age 41 and White participants, respectively. South Asian participants were less likely to be comfortable seeking medical care compared to White Participants (Table 6).

**Table 6:** Association of reported comfort with public health behaviors and demographic characteristics^1^.

**Statements**	**Gender**			**Age**					**Race**								
	**Female**	**Male**		**18–40**	**41–60**		**61**+		**White**	**Black**		**East** + **Southeast Asian**		**South Asian**		**Other**	
		**OR**	**CI**		**OR**	**CI**	**OR**	**CI**		**OR**	**CI**	**OR**	**CI**	**OR**	**CI**	**OR**	**CI**
Comfort with Mask Wearing	Reference	0.63	0.50–0.80^*^	Reference	1.63	1.24–2.15^*^	1.67	1.13–2.54^*^	Reference	0.55	0.39–0.78^*^	1.03	0.75–1.42	1.07	0.72–1.12	0.74	0.49–1.12
Comfort Seeking Medical Care		0.78	0.62–0.96^*^		2.15	1.67–2.78^*^	3.55	2.34–5.58^*^		0.71	0.52–0.99^*^	0.86	0.65–1.14	0.69	0.49–0.99^*^	0.87	0.59–1.30
Comfort Getting a COVID Test		1.08	0.88–1.32		1.46	1.17–1.82^*^	1.93	1.38–2.73^*^		0.60	0.45–0.81^*^	0.83	0.64–1.07	0.87	0.63–1.21	0.90	0.62–1.30

^1^Odds-ratio estimates of the response of agree/strongly agree with statements in the left column for different levels of the demographic variables.

^*^Denotes significant finding at α = 0.05.

## Discussion

We surveyed over 1,800 residents of Ontario, Canada between June–December 2020 to canvass perceptions and experiences of stigma during the COVID-19 pandemic. Thirty-nine percent of participants feared being stigmatized during the pandemic and 37% experienced stigmatization or racism during the pandemic. Moreover, 51% of respondents believed that racism had increased in Canada during the pandemic. Our findings should be viewed within the following context: the time period represents the end of the first and start of the second waves of COVID-19 in the province (20). Ontario remained in a state of emergency until July 29th 2020 following the first wave of COVID-19 in the country. In July 2020, mandatory public health measures, such as masking in public spaces, became widespread (23). September 2020 saw an increase in COVID-19 cases which led to new restrictions across the province, particularly in regions with higher case volumes (24, 25). At the end of our study period (December 2020), the province had broken the record for daily new infections and was entering a province-wide shutdown beginning December 26th (25, 26). To provide further context to the study period, Summer 2020 was referred to as the “summer of racial reckoning” (27, 28). Black Lives Matter marches, media reports reporting anti-Asian racism in Ontario cities, and calls for anti-racist strategies highlighted deep-rooted systemic inequities in Canada and worldwide (29–31).

The HSDF indicates that stigma drivers (e.g., fears, blame, stereotypes) and facilitators (cultural norms, policies) interact to result in stigma “marking” based on health conditions and/or demographic factors (18). In our survey, we observed stigma marking based on race/ethnicity (with significantly greater odds reported for East Asian participants), age (for both older and younger adults), and profession. Individuals who were racialized, aged 18–40, and Men were more likely to fear and report experiencing stigma compared to White individuals, individuals over 40, and Women, respectively.

Among the 22% of participants who experienced stigma because of their race/ethnicity, 58% were East or South East Asian, 15% were Black and 12% were South Asian as compared to 5% of White participants. Notably, East and Southeast Asian participants were 14.9 times more likely to report experiencing racial stigma as compared to White participants. Our results are consistent with the 2020 Statistics Canada Canadian Perspective Survey Series, which is a longitudinal survey study assessing Canadians' perceptions during the pandemic (9). The June 2020 survey revealed that 20% of Canadians surveyed feared being the target of unwanted acts or behaviors because of perceived exposure risk to COVID-19 (9). Of this 20%, 1 in 5 feared being stigmatized because of their race and 1 in 10 feared being stigmatized because they worked in healthcare. Further, 13% of those who feared stigmatization attributed it to their older age.

During the initial pandemic wave, individuals of Asian descent, specifically those of Chinese ethnicity, experienced significant stigmatization, racism and marginalization (32). The CCNC Toronto Chapter, a Chinese Canadian advocacy group and partner on this study, reported 1,150 cases of racist attacks submitted to their web platforms from March 2020 to February 2021 (33). Individuals reporting these attacks were predominantly adolescents and of East Asian descent (under 18 years); adults over 55 years were more likely to report being physically assaulted. Their study identified the impact of intersecting characteristics on racist attacks, with individuals who reported the attacks in Chinese (versus English) being 34% more likely to experience emotional distress and 100% more likely to be physically assaulted (33). Similar incidents have been observed in the United States. Gardner et al. reported that 23% of Asians living in the US experienced COVID-19-related discrimination due to perceptions of blame toward China for the pandemic (25). In turn, these sentiments led to workplace discrimination and hiring biases toward Asian Americans during the pandemic (34). Similar to the findings of the CCNC Toronto Chapter, a US coalition of Asian American advocacy groups reported over 1,500 instances of racism, hate speech, or attacks against Asians and Asian Americans attributed to stigmatizing language and statements by prominent politicians and on social media (35).

Of the 39% of participants in our study who feared being stigmatized during the pandemic, 24% were White and 60% were Women. Although White participants were less likely than racialized participants to fear being stigmatized based on race/ethnicity, other reported fears of stigmatization were attributed to age, political views, and place of work. In our study, participants did not perceive gender to be a significant factor that led to increased stigma, however, Men were more likely than Women to report fear and experiences of stigma based on sex, gender, immigration status, level of ability, sexual orientation, level of education, religion, place of work and political views. Reports from the CCNC also suggest that Men were more likely than Women to be physically assaulted during the pandemic (24). Intersectional analysis may provide further context to these results. For instance, one qualitative Brazilian study suggests Men who typically experience class or gender privileges were surprised by stigmatization they experienced after contracting COVID-19 (36). The authors suggested this subset of the population is not used to being treated impolitely or being discriminated against as compared to other groups and therefore may have a heightened awareness of COVID-related stigma (36). Additionally, emerging data suggest significant gender differences in perceptions of COVID-19. In a survey of over 20,000 participants between March–April 2020 from eight high-income countries, Galasso et al. identified Women as more likely to perceive COVID-19 as a serious public health threat and to comply with preventive public health measures (37). Similarly, the Statistics Canada Survey reports that among those who feared stigmatization, 50% said it was because they do not wear a mask or did not intend to wear a mask (notably, data were gathered prior to mandatory mask mandates). This sentiment was proportionally higher among Men, younger adults, and Canadian-born individuals (9). Additional research to explore the mechanisms by which gender differences (particularly in consideration of other intersecting factors) manifest into fear of stigmatization or willingness to participate in public health behaviors are warranted.

The impact of stigma on health behaviors, and subsequently health outcomes, has been widely reported (38–40). For instance, a recent meta-analysis pooled the prevalence of stigma in infectious disease epidemics (e.g., SARS, H1N1, Zika, COVID-19) and identified 34% prevalence across all populations (40). In our study, 74, 68, and 59% of participants were comfortable masking, seeking medical care if exposed to/sick with COVID-19, and getting tested for COVID-19, respectively. However, Men and participants 18–40 were less likely to report comfort masking and seeking medical care compared to Women and participants 40+ years, respectively. Black participants were less comfortable with all three behaviors and South Asian participants were less comfortable seeking medical care as compared to White participants. Longstanding, systemic inequities and mistreatment leading to a lack of trust in the healthcare system may explain these observed patterns by race/ethnicity (41–43). The HDSF may further provide insights on how intersecting stigma experiences, when coupled with stigma practices, can manifest into health behaviors, access, and subsequently, health related outcomes (10).

Finally, another important and perhaps unique factor experienced during the COVID-19 pandemic was the global public's demand for information (e.g., how is COVID-19 transmitted, treated) contrasted against the scientific community's limited knowledge of the disease, particularly in the early days of the pandemic. The gap between the public's desire for knowledge coupled with the scientific community's efforts to generate evidence led to waves of infodemics, which included stigmatizing messages as well as conspiracy theories that give direct rise to stigma (44, 45). Stigmatizing information was widespread on social and mainstream media. Editorial cartoons consistently “othered” groups of people, (particularly people of Chinese origin or descent), racist and exclusionary hashtags trended on Twitter, derogatory comments toward prominent Asian-Canadians circulated widely, and trending tweets to ban Chinese immigration and boycott Chinese-owned businesses in Spring 2020 impacted entire neighborhoods in Canadian cities (30, 31, 46, 47). This stigmatizing information led to misinformation which was often shared widely. In fact, a Statistics Canada study estimated that over half of Canadians share misinformation/ unverified information online without first confirming its accuracy (48). Additional research exploring the intersection between stigma and misinformation in the Canadian context is both warranted and underway (49).

Strengths of our study include use of an integrated knowledge translation approach, meaning the project was designed and conducted in partnership with advocacy organizations. Use of this approach ensured our data explored questions and concepts that were important to community partners and will further the Canadian discourse on the impact of stigmatization during the COVID-19 pandemic. Second, we aimed to recruit a sample that reflected the diversity of Ontario's population; this approach allowed us to conduct secondary analyses to explore perceptions by age, race/ethnicity, and gender and contributes to the emerging literature on the use of an intersectionality lens in the conduct of health services research. Finally, this study is part of a research program that explored fear, stigma and misinformation in Canada during the COVID-19 pandemic; our studies will aim to provide actionable recommendations to improve communication and messaging in future public health emergencies.

This study has limitations. Our survey was limited to residents of Ontario and may not be representative of experiences of individuals residing in other Canadian provinces and territories. Study participants' demographics were skewed toward Women (54%) and individuals aged 18–40 (54%). Fifty-percent of the sample was college educated, which is reflective of the Canadian population (50). Our survey was limited to the first and second waves of the pandemic; it is likely that experiences and perceptions of stigmatization continued to evolve throughout the pandemic. For instance, in 2021, sentiments towards vaccinations stigmatized both those who were and were not in support of mandatory vaccination policies (51–53). Additionally, our study did not explore the impact of health related stigmas; for example, we did not evaluate whether individuals with pre-existing health conditions (e.g., HIV), individuals with disabilities, and individuals with comorbidities experienced intersecting health-related stigmas. Our study did not comprehensively explore the impact of COVID-19 on individual or social characteristics and certain groups may have been overlooked in our research. For instance, studies have highlighted the disproportionate impact of the pandemic on gender-diverse individuals (who were underrepresented in our sample) or the impact of political ideologies on attitudes toward COVID-19 (53, 54). Finally, we did not perform interaction analyses to determine the impact of intersecting categories (e.g., age x gender x race/ethnicity) on stigma perceptions and experiences. Our survey contained ordinal scale questions, which challenged our ability to interpret odds-ratio estimates for our independent variables. Additionally, our approach to demographic data collection (e.g., age ranges rather than specific ages) did not allow for linear analyses. In future, similar studies can design survey questions in a manner that will facilitate such analyses (55).

## Conclusion

Our study describes fear and experiences of stigmatization during the COVID-19 pandemic among residents of Ontario, Canada. Racialized individuals, individuals aged 18–40 and Men were more likely to report fear and experiences of stigmatization during the pandemic. Understanding the factors that drive stigmatization during a health emergency and developing strategies to mitigate these sentiments are essential. Additional research on the impact of stigmatization during COVID-19 in Canada and whether it impacted health outcomes is needed.

## Data availability statement

The raw data supporting the conclusions of this article will be made available by the authors, without undue reservation.

## Ethics statement

This study received ethics approval from the Toronto Academic Health Science Network REB (REB#20-092). Written informed consent for participation was not required for this study in accordance with the national legislation and the institutional requirements.

## Author contributions

CF and SS were responsible for data conceptualization and methodology. CF, JC, and ST facilitated data collection. JC and BP conducted the statistical analyses. CF and JC drafted the manuscript. All authors read, edited, and approved the final manuscript.
