# Supplementary material for: Exploring Canadian perceptions and experiences of stigma during the COVID-19 pandemic
Source: Front Public Health. 2023 Mar 7;11:1068268. doi: 10.3389/fpubh.2023.1068268 (PMC10027913; doi:10.3389/fpubh.2023.1068268)
Supplement: Supplementary file 1 [file Data_Sheet_1.PDF]

Appendix A: COVID-19 Stigma and Fear Survey Questions

Developing strategies and tools to combat misinformation, fear and stigma in the wake of COVID-19 Thank you for taking the time to learn more about this study! Before agreeing to participate, it is important that you read and understand the following explanation of the proposed study procedures. The following information describes the purpose, procedures, benefits, and risks associated with the study. It also describes your right to refuse to participate or withdraw from the study at any time. To decide whether you wish to participate in this research study, you should understand enough about its risks and benefits to be able to make an informed decision. This is known as the informed consent process. Please make sure all your questions have been answered to your satisfaction before participating in the online survey.

Background & Purpose of Research: This research study is being conducted by researchers at the Knowledge Translation Program, St. Michael's Hospital, Unity Health Toronto in partnership with the Chinese Canadian National Council for Social Justice (CCNC-SJ) and the Chinese Canadian National Council Toronto Chapter (CCNCTO), the Yee Hong Centre for Geriatric Care, the Singapore Geriatric Education & Research Institute (GERI) team, and research teams at Université Laval, University of Ottawa, and University of Toronto. We are aiming to create tools to combat misinformation, stigma and fear in response to the COVID-19 outbreak. We will aim for these strategies and tools to be both relevant to the Canadian context and generalizable to an international audience. To date, we have conducted interviews with members of the public, healthcare providers and policymakers to explore their perceptions and reactions to COVID-19. To supplement findings from the interviews we will be distributing an online survey to members of the public across Ontario.

Description of the Research: If you choose to participate, you will be asked to participate in an online survey. The survey should take approximately **30 minutes** to complete. Questions in the survey will explore your perceptions and reactions to the novel coronavirus or COVID-19 outbreak, as it relates to stigma, fear and misinformation. At the end of the survey there will be a few demographic questions to complete (e.g., age, gender, province where you reside, and race/ethnicity etc.). Responses to these questions are completely voluntary and are meant solely to help us in examining how different groups of people feel about the outbreak.

Potential Harms (Injury, Discomforts or Inconvenience): There are no known harms associated with this study. **Potential Benefits:** You may not experience any personal benefits from taking part in the study. However, you will have an opportunity to shape the development of KT strategies and tools to combat stigma and misinformation in response to COVID-19 in Canada. **Potential Costs and Reimbursement:**
Upon completion of the survey, you will be given the option to enter a draw for a chance to win one of the following: **1 $100 Visa gift card, 2 $50 Visa gift cards, and 5 $20 Visa gift cards.** Should you decide to enter the draw, you will taken to a separate screen to complete a form to enter the draw. The information you provide will not be linked to your survey response.

Privacy & Confidentiality: Completion of this survey is anonymous and completely **confidential**. All survey results will be stored on the Qualtrics website until downloaded onto our secure server at St. Michael's hospital for analysis. Qualtrics is a secure online data collection system. Their servers are protected by high-end firewall systems, and scans are performed regularly to ensure that any vulnerabilities are quickly found and patched. Data is stored in a specific Canadian location; it does not float around in the "cloud." In addition, all data are processed in that location, and are not moved to another jurisdictional area (e.g., outside Canada). Study personnel will ensure that all files are encrypted and password protected. All information collected will be stored for seven years. No information identifying you will be transferred outside the study team unless required by law. **Your data will not be shared with any other researchers or organizations for any reason.** The Unity Health Toronto Research Ethics Board may look at the study information collected for the purpose of monitoring the study. Data will be retained for no more than 7 years. It is important to understand that despite these protections being in place, there continues to be the risk of unintentional release of information. The principal investigator and study team at St. Michael's Hospital will protect the study records and keep all the information confidential to the greatest extent possible. The chance that this information will be accidentally released is minimal.

Participation and Withdrawal: Your participation in this study is voluntary. You can choose to not participate or you may withdraw at any time. Your participation, or choice to not participate, will not affect your current and future care or relationships at Unity Health Toronto. If a question is not applicable to you or you feel uncomfortable answering please complete them to the best of your ability. We would appreciate your responses to all questions, none of the individual questions are mandatory. Due to the anonymous nature of the survey (e.g. there is no way to link your name to the responses), you cannot retract your survey once you have submitted your responses.

Intro **Publication of research findings:** The results of this study may be presented at conferences, seminars or other public forums, and published in journals. We will publish our results in aggregate form only (this means that no identifying information will be included anywhere). The Unity Health Toronto Research Ethics Board may request to look at the study information collected for the purpose of monitoring the study. **Study Results:** You may be provided with a copy of the study report to review upon request. **Research Ethics Board Contact:** If you have any questions regarding your rights as a research participant, you may contact the Chair of Unity Health Toronto's Research Ethics Board at (416) 864-6060 ext. 2557 during business hours. **Study Team Members**

Sharon E. Straus, MD, FRCPC Principal Investigator, Director of Knowledge Translation Program, St. Michael's Hospital, Unity Health Toronto and Associate Professor, University of Toronto Tel: (416)

864-3068 (available Monday to Friday 9:00am – 5:00pm)
Christine Fahim, PhD Research Scientist
Knowledge Translation Program, Li Ka Shing Knowledge Institute, St. Michael's Hospital, Unity Health Toronto Tel: 416-864-6060 ext 77527 (available Monday to Friday 9:00am – 5:00pm) Email: christine.fahim@unityhealth.to

Christine Marquez, BSc Research Coordinator
Knowledge Translation Program, Li Ka Shing Knowledge Institute, St. Michael's Hospital Tel: 416-864-6060 ext. 77523 (available Monday to Friday 9:00am – 5:00pm) Email: christine.marquez@unityhealth.to
Jeanette Cooper, MSc Research Coordinator Knowledge Translation Program, Li Ka Shing Knowledge Institute, St. Michael's Hospital Tel: 416-864-6060 ext. *77507* (available Monday to Friday 9:00am – 5:00pm) Email: Jeanette.cooper@unityhealth.to

Anupa (Jyoti) Prashad, MScHSEd
Research Coordinator Knowledge Translation Program, Li Ka Shing Knowledge Institute, St. Michael's Hospital Tel: 416-864-6060 (available Monday to Friday 9:00am – 5:00pm) Email: anupa.prashad@unityhealth.to
Suvabna Theivendrampillai, BSc Research Assistant
Knowledge Translation Program, Li Ka Shing Knowledge Institute, St. Michael's Hospital
Tel: 416-864-6060 (available Monday to Friday 9:00am – 5:00pm)
Email: suvabna.theivendrampillai@unityhealth.to

If you should have any questions, concerns or technical difficulties, please do not hesitate to contact research assistant (Suvabna Theivendrampillai, suvabna.theivendrampillai*@unityhealth.to)* Please click here to download a PDF copy of this information sheet for your records. **Your consent to participate in the survey is demonstrated by your voluntary completion and submission of this survey. If you would like to continue, please click the "Next" button to continue to the survey If you do not wish to participate or continue please exit the browser.**

1. Are you currently a resident of Ontario?

- ○ Yes (1)
- ○ No (2)

2. What is your greatest fear or concern as related to COVID-19? Select all that apply

	Unsure (1)	I am not afraid/concerned (2)	I am a little afraid/concerned (3)	I am very afraid/concerned (4)	Not applicable (5)
Food security (1)	○	○	○	○	○
Access to consumer goods like hand soap and toilet paper (2)	○	○	○	○	○
Afraid someone in immediate family would become infected (3)	○	○	○	○	○
Personally afraid of becoming ill with COVID-19 (4)	○	○	○	○	○
Financial concerns (5)	○	○	○	○	○
Impact to the economy (6)	○	○	○	○	○
Lack of treatment; no vaccine (7)	○	○	○	○	○
Job loss, job insecurity (8)	○	○	○	○	○
Stigma/racism (9)	○	○	○	○	○

Blame for the virus/spreading the virus (10)	○	○	○	○	○
Other: please specify (11)	○	○	○	○	○

3. Do you think there is someone to blame for COVID-19?

○ Not at all (1)

○ A little (2)

○ Somewhat (3)

○ A lot (4)

○ Quite a lot (5)

Display This Question:

If Do you think there is someone to blame for COVID-19? = A little

Or Do you think there is someone to blame for COVID-19? = Somewhat

Or Do you think there is someone to blame for COVID-19? = A lot

Or Do you think there is someone to blame for COVID-19? = Quite a lot

4. Who do you blame for COVID-19? List

____

5. Please rank the following statements about COVID-19 and stigma:

	Not at all (1)	A little (2)	Somewhat (3)	A lot (4)	Quite a lot (5)
I think fear and misinformation around COVID-19 has been targeted towards certain groups. (1)	○	○	○	○	○
I think people of certain races/ethnicities have been stigmatized because of COVID-19. (2)	○	○	○	○	○
I think young adults have been stigmatized because of COVID-19. (3)	○	○	○	○	○
I think older adults have been stigmatized because of COVID-19. (4)	○	○	○	○	○
I think healthcare workers have been stigmatized because of COVID-19. (5)	○	○	○	○	○
I think other essential workers have been stigmatized because of COVID-19. (6)	○	○	○	○	○
I think people have been stigmatized during COVID-19 because of their gender. (7)	○	○	○	○	○
I think people have been	○	○	○	○	○

stigmatized during COVID-19 because of their political/ideological beliefs. (8)

6. Please rate the following statements on a scale of strongly agree to strongly disagree.

	Strongly disagree (1)	Disagree (2)	Neither agree nor disagree (3)	Agree (4)	Strongly agree (5)
I have been stigmatized during COVID-19 because of my sex (1)	○	○	○	○	○
I have been stigmatized during COVID-19 because of my gender (2)	○	○	○	○	○
I have been stigmatized during COVID-19 because of my race/ethnicity (3)	○	○	○	○	○
I have been stigmatized during COVID-19 because of my age (4)	○	○	○	○	○
I have been stigmatized during COVID-19 because of my citizenship/immigration status (5)	○	○	○	○	○
I have been stigmatized during COVID-19 because of my disability/level of ability (6)	○	○	○	○	○
I have been stigmatized during COVID-19 because of my sexual orientation (7)	○	○	○	○	○
I have been stigmatized during	○	○	○	○	○

COVID-19 because of my level of education (8)					
I have been stigmatized during COVID-19 because of my religion/religiosity (9)	○	○	○	○	○
I have been stigmatized during COVID-19 because of my political view/identity (10)	○	○	○	○	○
I have been stigmatized during COVID-19 because of my occupation/place of work (11)	○	○	○	○	○

7. Please rate the following statements on a scale of strongly agree to strongly disagree.

	Strongly disagree (1)	Disagree (2)	Neither agree nor disagree (3)	Agree (4)	Strongly agree (5)
I fear being stigmatized during COVID-19 because of my sex (1)	○	○	○	○	○
I fear being stigmatized during COVID-19 because of my gender (2)	○	○	○	○	○
I fear being stigmatized during COVID-19 because of my race/ethnicity (3)	○	○	○	○	○
I fear being stigmatized during COVID-19 because of my age (4)	○	○	○	○	○
I fear being stigmatized during COVID-19 because of my citizenship/immigration	○	○	○	○	○

status (5)					
I fear being stigmatized during COVID-19 because of my disability/level of ability (6)	○	○	○	○	○
I fear being stigmatized during COVID-19 because of my sexual orientation (7)	○	○	○	○	○
I fear being stigmatized during COVID-19 because of my level of education (8)	○	○	○	○	○
I fear being stigmatized during COVID-19 because of my religion/religiosity (9)	○	○	○	○	○
I fear being stigmatized during COVID-19 because of my political view/identity (10)	○	○	○	○	○
I fear being stigmatized during COVID-19 because of my occupation/place of work (11)	○	○	○	○	○
I do not fear being stigmatized during COVID-19 (12)	○	○	○	○	○

8. I fear being stigmatized during COVID-19 because:

☐ I do not wear a mask all the time (1)

☐ I do not socially distance all the time (2)

☐ I have a trip/vacation booked out of the country (3)

☐ I have health conditions that cause me to cough or sneeze (4)

☐ I have had COVID-19 (5)

☐ Someone I am close with has had COVID-19 (6)

☐ Other (7) ____

9. Please rate the following statements on a scale of strongly agree to strongly disagree.

	Strongly disagree (1)	Disagree (2)	Neither agree nor disagree (3)	Agree (4)	Strongly agree (5)
I have felt/would feel comfortable wearing a mask in public (1)	○	○	○	○	○
I have felt/would feel comfortable getting tested for COVID-19 (2)	○	○	○	○	○
I have felt/would feel comfortable seeking medical care if I felt sick (3)	○	○	○	○	○
I have experienced a slur/joke about my identity during COVID-19 (4)	○	○	○	○	○
I have experienced rejection because of my beliefs about masking or social distancing during COVID-19 (5)	○	○	○	○	○
I believe racist views have increased towards certain	○	○	○	○	○

racial/ethnic groups in Canada during COVID-19 (6)					
I believe racist views have decreased towards certain racial/ethnic groups in Canada during COVID-19 (7)	○	○	○	○	○
People have acted as if they were afraid of me during COVID-19 (8)	○	○	○	○	○
People have acted as if I am not smart during COVID-19 (9)	○	○	○	○	○
People have threatened or harassed me during COVID-19 (10)	○	○	○	○	○
People said things that were untrue about my race/ethnic group related to COVID-19 (11)	○	○	○	○	○
People have treated me poorly at restaurants/stores during COVID-19 (12)	○	○	○	○	○
I believe Canadians are speaking out against discrimination and stigmatization (13)	○	○	○	○	○

10. Do you have any other feedback, comments, or concerns regarding stigma, fear, or misinformation surrounding COVID-19 that you would like to share with us?

11. Which best describes your current gender identity?
Note: these questions are for demographic purposes only, we will NOT identify you based on your answers; your answers will remain confidential; your data WILL NOT be shared with anyone

- ◯ Male (1)
- ◯ Female (2)
- ◯ Indigenous or other cultural gender identity (e.g., two-spirit) (3)
- ◯ Other (e.g., gender fluid, non-binary) : (4) ____
- ◯ Prefer not to answer (5)

12. What is your current age?

- ◯ 18-30 (1)
- ◯ 31-40 (2)
- ◯ 41-50 (3)
- ◯ 51-60 (4)
- ◯ 61-70 (5)
- ◯ 71-80 (6)
- ◯ 80+ (7)

13. Do you identify as an Indigenous person?

- ◯ Yes (1)
- ◯ No (2)

Display This Question:

If Do you identify as an Indigenous person? = Yes

14. Please select the Indigenous Identity:

- ◯ Non-indigenous (1)

○ Metis (2)

○ First Nations (3)

○ Inuit (4)

○ Other (5) ____

15. Do you identify as a 'racialized person'? (Defined as *persons in Canada, other than indigenous peoples, who are non-Caucasian in race or non-white in colour, regardless of place of birth or citizenship*)

○ Yes (1)

○ No (2)

○ Prefer not to answer (3)

16. Which of the following best describes your race or ethnic group? Please check all that apply.

☐ Black – African (1)

☐ Black – North American (2)

☐ Black – South and Central American (3)

☐ Black - Afro-Carribean (4)

☐ Black - Afro-European (5)

☐ LatinX or Hispanic - Central American (6)

☐ LatinX or Hispanic - South American (7)

☐ LatinX or Hispanic - Carribean (8)

☐ LatinX or Hispanic - European (9)

☐ East Asian (10)

☐ Southeast Asian (11)

☐ South Asian (12)

☐ Middle Eastern - North African (13)

☐ Middle Eastern - Middle Eastern/West Asian (14)

☐ White – European (15)

☐ White - North American (16)

☐ South American (17)

☐ Other (please specify): (18)

____

There are a few additional demographic questions. Please NOTE that all responses are anonoymous.

17. What language(s) do you speak?

____

18. What is your current employment status? Check all that apply to your current situation.

- ☐ Full-time (1)
- ☐ Part-time (2)
- ☐ Retired (3)
- ☐ Caregiver (4)
- ☐ Student (5)
- ☐ Seeking work (6)
- ☐ Other (i.e. receiving CERB, disability benefits, etc.) (7) ____

19. What is the highest level of education you have completed?

- ○ 12th grade or less (1)
- ○ Graduated high school or equivalent (2)
- ○ Some college/university, no degree (3)
- ○ College/University degree (4)
- ○ Post-graduate degree (5)

20. What is your immigration status? What are the first

- ○ Canadian citizen (born in Canada) (1)
- ○ Canadian citizen (foreign born) (2)
- ○ Permanent resident (3)
- ○ Temporary resident/student visa (4)
- ○ Other (5) ____

21. What are the first 3 digits of your postal code?

Note, this is for demographic purposes only, we will NOT ask for your address and you will NOT receive any correspondence from us. Our data will not be shared with anyone.

____
